# Extracts from the Records of the Boston Society for Medical Improvement

**Published:** 1859-07

**Authors:** F. E. Oliver

**Affiliations:** Secretary


### EXTRACTS FROM THE RECORDS OF THE BOSTON SOCIETY FOR MEDICAL IMPROVEMENT.

BY F. E. OLIVER, M. D., SECRETARY.

APRIL 11th.—*Malignant Disease of the Upper Maxillary Bone.* Dr. J. MASON WARREN reported this and the three following cases:

"In February, 1857, I was called to see a lady, about 65 years of age, affected with a malignant tumor of the upper maxillary bone. Her account of the origin of it was this: About a year previous, as she was attempting with a hammer to draw out a nail from the wall, the hammer slipped, and she received a blow from the handle of it on her face, just under the left infra-orbitar foramen. A great deal of inflammation followed the blow, with pain and constitutional symptoms, which continued five or six weeks. The inflammation having subsided, she felt a constant degree of tension in her face. The alveolar processes seemed gradually to expand, the gums assumed a fungous appearance, and the teeth on that side either dropped out, or were extracted by the fingers. At this time an elastic tumor showed itself in the mouth, which finally burst, and when I first saw her a gangrenous mass projected from the opening. The patient had a very quick pulse, her appetite was extremely small, if not entirely lost, and she seemed to her friends in a rapidly failing state. I immediately proceeded to clear away the slough from the mouth, which was fortunately effected without any hemorrhage, and gave her a wash, with the object of destroying the putrid emanations from the wound. Quinine and wine were administered, and very soon her appetite was sufficiently restored to take nourishing food.

"In the course of about three weeks her health was so much improved that it was thought expedient to bring into question what surgical procedure, if any, could be adopted for the removal of the tumor. The pain still continued at times to be excessive, extending over the face, and was of what is called a neuralgic character.

"Having requested Drs. Townsend and Cabot to see the patient in consultation, we agreed that the tumor was of a malignant nature; but as it was still limited to one of the maxillary bones, and had not yet implicated the surrounding parts, while the pain was so severe as to require some active surgical interference, we decided, notwithstanding the danger of a recurrence, that it was proper to advise an operation. The patient was disposed at first to accede to our advice, having the nature of the case fairly laid before her, but after a day's consideration and conversation with her friends, she said that if I thought there was the slightest danger of a recurrence, she was unwilling to submit to it.

"The subsequent history of the case is as follows: The tumor gradually crept along the floor of the mouth, invading the opposite maxillary bone. Projecting into the mouth, it opened at numerous points, obstructing the passage to the fauces, and almost poisoned the patient with foul secretions. The pain, during all this time, in the superior maxillary nerve, was excessive, requiring the most powerful internal and external medicines. It may perhaps be well to mention the following occurrence as instructive:

"During one of her violent paroxisms of pain, she requested an attendant to give her a teaspoonful of medicine from a phial supposed to contain a solution of morphine. By mistake, a teaspoonful of a strong solution of aconite, only used for external application, was administered; and the attendant, not being satisfied with its effects, very shortly administered a second dose. The aconite very soon began to show its specific effects, and the mistake was discovered. Dr. J. F. W. Lane, who had formerly attended the family, and who lived in the vicinity, was called. Dr. Lane at once administered a very stimulating emetic, and, following this by other very active measures, after a number of hours was able to leave her in a safe condition. The effects of the aconite on the brain showed themselves for a week or ten days afterward.

"This patient died in a very suffering state about six months after I first saw her, the end being finally accelerated by repeated hemorrhages from the sloughing mass."

*Removal of the Upper Maxillary Bone.*—"In the summer of 1857, I was requested by Dr. Reynolds to visit with him, in consultation, a patient who was suffering from an affection of the left upper jaw-bone. Some months previously, the trouble had commenced by an irritation in the neighborhood of the left lachrymal passage, which produced an obstruction in the passage, and a discharge of tears over the face. This was followed by a distended feeling in the upper maxillary bone, which gradually increased, and finally terminated by the appearance of an aperture in the alveolar process of one of the molar teeth, which discharged a quantity of blood and gave relief to the patient.

"When I first saw her, there was the appearance in the left nostril of a polypoid tumor, and she had suffered from one or two bleedings from this situation. After carefully investigating the previous symptoms, a probe was passed into the opening in the mouth, which penetrated deep into the maxillary sinus, and was followed by a free discharge of blood. The patient being rather low in health, and proposing to make a visit to her friends in Maine, I provided her with instructions, and she agreed to see me again in three or four weeks.

"At the expiration of the time appointed, she returned to Boston, and I found that the treatment had produced a decided improvement in her general health. The tumor in the nostril had increased, as well as the distension about the maxillary sinus, and she had suffered from one or two pretty severe hemorrhages. In the course of the following week, a bleeding of so severe a nature took place as to render it necessary to have some active surgical procedure at once adopted.

"Before making up my decision, I passed a finger into the nostril, which disclosed a large opening into the maxillary sinus, from which the tumor into the nose seemed to have projected. The jaw in the neighborhood of the aperture in the mouth had, since the last examination three weeks before, been more or less forced downward into a rounded elastic tumor. These circumstances being considered, seemed to leave but little doubt that the maxillary sinus was occupied by a tumor, which was gradually forcing itself out from the bony cavity in which it had originated. I advised, therefore, the extirpation of the superior maxillary bone as the only means likely to eradicate the disease, to which she at once consented. Having no accommodations in the hotel where she was staying, for an operation of such importance, she took a private room at the Hospital, where the following operation was performed:

"The patient, sitting in a chair with her head supported, was moderately etherized with sulphuric ether. An incision was made, commencing near the external angle of the eye, and terminating at the angle of the mouth; the cheek was then rapidly dissected up. The bone having now been made clear from the soft parts, the sponge, well-charged with ether, was again placed over the face, the flap being held up by an assistant. This served to etherize the patient by a second dose, and at the same time by its rapid evaporation to stop the effusion of blood from the small vessels, so that no blood penetrated the patient's mouth or fauces. The maxillary bone was now sawn through at the external part of the orbit, and the malar bone divided by the cutting forceps, as well as the nasal process of the maxillary. A large pair of Liston's forceps, double the size of those in ordinary use, which was made for me under the direction of that distinguished surgeon, was passed into the mouth and nostril, and divided the bone with as much ease as if cutting a piece of paper. A sharp-pointed knife was now drawn across the palate, opposite the suture of the palatine and maxillary bones, and the mucous membrane covering the palate cut through. The jaw was seized by a strong pair of double-hooked forceps, and the whole mass depressed, the superior maxillary nerve, as it passed along the floor of the orbit, being cut off. The remaining soft parts were next divided by curved scissors, and the operation terminated, the os palati, with the soft palate of the same side, not being interfered with. The whole operation lasted about ten minutes. The hemorrhage was not excessive, and the vessels were easily secured. The edges of the wound were at once approximated by sutures, and a bit of lint moistened with cold water laid over the surface.

"No lint, bits of sponge, or other substances, as recommended by some of the French surgeons for filling up the cavity made by the removal of the jaw, were used in this or the other cases in which I have done this operation. Whenever I have seen them used, they have been the source of much irritation, have been with difficulty removed, and the cause of a most offensive odor from the retention of the foul secretions in the mouth.

"The only fact worthy of mention in the subsequent history of the case, was the occurrence, at the end of about a week, of a hemorrhage from the interior of the wound, which, although it gave rise to some alarm to the patient, was easily arrested by careful plugging with a sponge. She recovered fully and entirely, and now, at the end of nearly two years, I have heard of her in the enjoyment of good health. The eye suffered no injury from the operation.

"The tumor was of a fibrous character, and was completely bounded by its capsule. In its expansion it had at first nearly obliterated the lachrymal passage, next it had produced an absorption of the bone in the vicinity of the nostril, forcing its way through into that cavity, and finally it was making its way downward through the bone into the back part of the mouth.

"The operation was as effectual and satisfactory in its results as any one of this description that I have ever done or witnessed."

*Removal of the Upper Maxillary Bone.*—"Mrs. G., aged 49 years, applied to me in September, 1857, for a tumor of the left upper jaw-bone. She was a small thin woman, of a delicate constitution, and somewhat sallow complexion. Her health was moderately good with the exception of a tendency to rheumatism, having three times had rheumatic fever. She knew of no disposition to hereditary cancer. She was the mother of several children.

"Three years previously she perceived a fullness in the cheek-bone, and at the same time there was a slight and constant discharge from the nostril of the same side. This discharge continued and the swelling gradually increased until July of the present year, when she suffered so much uneasiness from it, that her physician punctured the antrum after having extracted a tooth. This, at the time, was followed by a small discharge of blood; but three days afterward a copious discharge of pus, as she says, took place, which has continued since in varying quantities. When the discharge is small, there is much fullness and pressure about the antrum, which is relieved by an increase of the flow.

"When I first saw this patient, the whole upper jaw-bone seemed to be enlarged. The tumor had not made its way out into the mouth, but seemed disposed to do so into the cheek, the integuments of which were somewhat reddened and a little œdematous. I advised the patient to an operation, informing the friends of the probable nature of the disease and likelihood of a recurrence, but at the same time that an operation offered the only chance for her relief; that the disease would soon come to an open ulcer on the face, and she would die a lingering, painful and disgusting death; that the operation, though a very formidable one in appearance, was not actually so dangerous as many of the important operations, which were done to save life, and that the pain could be completely prevented. After this representation she at once consented.

"The operation was performed in almost precisely the same manner as the one just before detailed, the palatine bone and palate being preserved. In depressing the bone after its attachment had been divided, a portion at its posterior part was found adherent, and was left attached to the pterygoid process so as afterward to require removal by the chisel. This circumstance I have once, or twice, seen happen in removal of the superior maxillary bone, the natural adhesion of the part being almost increased to anchylosis by the inflammatory action, which had been going on in its neighborhood. It is of so frequent occurrence, that it might be well in every case, as recommended by Dr. J. C. Warren, to pass a chisel behind the bone, and loosen it by two or three blows of the mallet.

"This patient had a very good recovery, and returned home about three weeks after the operation, in good health and spirits. She continued well for a time, but has, I believe, since had a return of the disease."

*Excision of the Upper Jaw.*—"April, 1859. Mrs. N. M., a small thin woman, 44 years of age, and mother of seven children, with no hereditary taint that she was aware of, had always enjoyed good health till five months ago, when a few weeks before the birth of her last child, which is now four months old, she was seized with a pretty sharp pain in the right upper maxillary bone. This troubled her more or less until the birth of the child, which was natural, and which she was able to nurse up to the time of her application to me for advice. About two months since, her face began to swell, and what was supposed to be a collection of matter took place between the upper lip and the alveolar process. This was punctured by a physician, but only blood issued at first, though she said there was, a few days after, a discharge of matter. Her face has continued to swell from that time, and now presents the following appearances:

"A tumor seems to have possession of nearly the whole of the upper jaw-bone. On the inside of the mouth, the palate is pressed down by it, and does not reach quite up to the median line. External to the alveolar process, the teeth having all dropped out, the tumor extends from the root of of the canine tooth as far back as the last molar. On the face, the swelling extends quite back to the ear, but the root of the zygomatic process can be felt on very firm pressure, as if part of the swelling might be owing to serous infiltration. The whole of the lower bony margin of the orbit is lost, and its place supplied by an irregular tumor. The pupil of the eye is turned upward, though by closing the other eye, and by an effort, the patient has the power of bringing it into proper position, the sight being somewhat enfeebled. No tumor is to be perceived in the nostril, though there is occasionally a bloody discharge from it. The skin over the whole of the tumor is movable, but somewhat tense and glossy. The digestive organs are in good order, and the pulse is not materially affected. Excessive pain in the swelling, of a bursting, insupportable character, together with a very rapid increase of the tumor, induced her to come to Boston for advice, though she was obliged to do so at the expense of giving up nursing her infant.

"The tumor appeared to me, without any doubt, of a malignant character, and was so considered by my colleagues at the Hospital. We advised an operation as a method of relief, although, from the very rapid increase of the disease, we could not think it offered much chance of a permanent cure. This being stated to the patient and her husband, she determined to have it done, as she could not longer support the suffering from the disease.

"The incisions are made a little different form those I have hitherto done, on account of the extension of the disease so far backward. The first incision commenced midway between the orbit and auditory passage, and extended in a semicircular form to the angle of the mouth, with a very broad backward sweep, instead of commencing, as in the other cases, just back of the orbitar process of the superior maxillary bone. All the incisions in the bone were made with Liston's large forceps, which cut through it with the greatest ease. Some difficulty was found in depressing the tumor, on account of the degeneration of the bone about the orbit, which would not allow a firm hold to be taken with the hooked forceps, so that it was necessary to seize and depress it with the fingers. After the removal of the tumor, it was found that some of the cancer had insinuated itslf into the pterygoid fossa; this was scooped out clean with the fingers, and a hot iron applied to the neighborhood. There was not much arterial bleeding, and but one artery required ligature, although in the course of the operation there was a universal venous hemorrhage from the tumor, as is often observed in cases of operation for malignant disease. The patient during the whole time was fully under the influence of ether, and blood was prevented from running down into the trachea by care, position and constant sponging of the fauces. A number of sutures were introduced, preparatory to closing the wound, which, however, was left open for two hours, exposed to the air, until all danger of bleeding had ceased, when the edges were nicely adjusted, and a compress wet with cold water laid over.

"I saw the patient in the afternoon, found her quite easy, relieved from the pain she had previously suffered, and able to swallow liquids, though with some difficulty. The eye regained its natural position as soon as the principal tumor had been taken from it, and she was altogether much more comfortable than could have been antitipated in a feeble person so soon after so severe an operation.

"No unpleasant symptoms followed the operation, the wound in the cheek healing by the first intention, and she was able to leave the Hospital, to return home, in a fortnight. A small abscess of the cheek required opening the day she left."—*Bost. Med. & Surg. Jour.*

